# Initial Evaluation by a Non-Surgeon Provider Does Not Delay the Surgical Care of Pediatric Forearm and Elbow Trauma in a Walk-In Orthopaedic Clinic

**DOI:** 10.7759/cureus.8139

**Published:** 2020-05-15

**Authors:** Matthew Fournier, Robert Neel, David Spence, Jeffrey Sawyer, Benjamin Sheffer, Derek Kelly

**Affiliations:** 1 Orthopaedic Surgery and Biomedical Engineering, University of Tennessee-Campbell Clinic, Memphis, USA; 2 Orthopaedics, University of Tennessee Health Science Center, Memphis, USA; 3 Orthopaedic Surgery, University of Tennessee, Memphis, USA

**Keywords:** pediatric trauma, walk-in, nonoperative providers, delays in care

## Abstract

Introduction

Walk-in and after-hours clinics are being increasingly utilized in orthopedics and are especially beneficial for patients with simple sprains, fractures, or overuse injuries that might otherwise require an emergency room visit. To meet the increased patient load, additional staffing often is required, which might include a family medicine physician, nurse practitioner, or physician assistant. Few studies have evaluated the performance of these non-surgeon providers in an orthopedic clinical setting. This study compared the time to definitive care of pediatric patients with forearm and elbow injuries between non-surgeon providers in a walk-in clinic, orthopedic surgeons in a walk-in clinic, and a pediatric orthopedic surgeon in a regular clinic.

Methods

Children who had closed reduction and fixation of an elbow or forearm injury from January 2010 to December 2017 were identified. The patients were divided into groups: patients initially evaluated in a walk-in clinic by a non-surgeon provider; patients initially evaluated in a walk-in clinic by an orthopedic surgeon; and patients initially seen by a fellowship-trained, pediatric orthopedic surgeon in a regular clinic (control group). Neither type of provider (non-surgeon or surgeon) in the walk-in clinics definitively treated any injury but rather transferred care of the patient to a pediatric orthopedic surgeon. The number of clinic visits until surgery, the number of providers seen, the days before evaluation by a pediatric orthopedic surgeon, and the number of days before definitive surgical treatment were documented.

Results

Of the 162 patients identified, 36 (22%) were initially seen by an orthopedic surgeon and 62 (38%) by a non-surgeon provider in a walk-in clinic. The remaining 64 (40%) (control group) were initially seen in a regular office visit by a pediatric orthopedic surgeon. There were no significant differences noted for patients treated by orthopedic surgeon and non-surgeon providers in days before a referral visit to the pediatric orthopedic surgeon (3.7 vs. 3.9, respectively; p = 0.63) or days to surgery for definitive treatment (5.2 vs. 4.8, respectively; p = 0.62). The average number of providers seen (1.58 vs. 1.63, respectively; p = 0.69) and average number of clinic visits before surgery (2.08 vs. 2.06, respectively; p = 0.76) also were similar when comparing the two groups. The control group had significantly fewer days from evaluation to surgical treatment than the surgeon walk-in group (3.3 days vs. 5.2 days, p < 0.05) and the non-surgeon walk-in group (3.3 days vs. 4.8 days, p < 0.05).

Conclusion

There was no difference in the number of days to transfer patient care to a pediatric orthopedic surgeon between non-surgeon providers and orthopedic surgeons in the walk-in clinic. However, there was a one-day delay reaching definitive treatment when initial evaluation occurred in a walk-in clinic, regardless of whether the patient was initially seen by a surgeon or non-surgeon, when compared to an initial evaluation by a pediatric orthopedic surgeon.

## Introduction

Providing pediatric orthopedic care to patients in the current healthcare climate commonly involves practitioners with a variety of backgrounds and training. The surgeon leads the healthcare team, but this team may be augmented by family medicine providers, nurse practitioners, or physician assistants [1-3]. The goal of any orthopedic practice is to provide high-quality, efficient care that effectively addresses the demands of a patient population and utilizes available resources appropriately.

Complaints of in-toeing, overuse injuries, and simple fractures comprise a significant proportion of services provided in a pediatric orthopedic clinic, but these complaints may not always require evaluation by a surgeon to be treated safely and effectively [3,4]. In a scenario in which a practice has increased demand for outpatient clinical or clerical duties, it may not be feasible to meet this demand by hiring another pediatric orthopedic surgeon. Many practices also support an “after-hours” triage clinic that may operate outside conventional business hours and on weekends [3,5]. It may be beneficial in these circumstances to bolster the number of providers seeing patients in this clinic without placing undue burden on already busy surgeons. This role can potentially be filled by non-surgeon providers for musculoskeletal care, maximizing the number of patients seen during regular clinic hours while minimizing financial burden on the practice.

Despite the significant role that non-surgeon providers already play in many orthopedic practices, there is little objective evidence concerning their performance in clinical settings compared to orthopedic surgeons [1-3,6-9]. In many cases, transfer of care from a non-surgeon provider to a surgeon is necessary should an injury requiring surgery be identified by the non-surgeon provider. This transfer of care may represent one potential opportunity for delay in the treatment of patients requiring surgery [9].

The purpose of this study was to compare the plan of care between non-surgeon providers and orthopedic surgeons in a walk-in clinic in regard to the number of provider visits required before surgery, the number of providers seen during the episode of care, the time until the first office visit with a pediatric orthopedic surgeon, and the number of days to surgery for treatment of pediatric elbow or forearm injuries. We hypothesized that initial evaluation by non-surgeon providers in a walk-in clinical setting would not significantly delay treatment.

## Materials and methods

This retrospective cohort study was approved by our Institutional Review Board (IRB no. 16-05056-XP). Pediatric patients (younger than 18 years of age) who were evaluated at one institution from January 2010 to December 2017 were identified in the electronic medical records system using the Current Procedural Terminology (CPT) code for elbow or forearm injury. The patients were subsequently placed into three groups: patients who were seen by a non-surgeon provider in a walk-in clinic; patients who were seen by an orthopedic surgeon in a walk-in clinic; and patients who were seen by a fellowship-trained pediatric orthopedic surgeon at a scheduled appointment (control group). Orthopedic surgeons in the walk-in clinic included fellowship-trained providers from a variety of subspecialties as well as current orthopedic fellows at the study institution. Non-surgeon providers included family medicine and physical medicine physicians, nurse practitioners, and physician assistants. All patients with injuries requiring surgical treatment were transferred to a pediatric orthopedic surgeon for definitive care. Patients in the control group were initially evaluated and subsequently underwent management by one fellowship-trained pediatric orthopedic surgeon. These patients had been scheduled for a regular appointment with the pediatric provider and scheduled for procedural or surgical care after that visit by the same provider.

All providers were employed by the same orthopedic practice, and only patients who were evaluated on a walk-in basis, without a scheduled appointment or prior evaluation of the injury in question, were included in the study. Only acute injuries, defined as having occurred within seven days of presentation, were included. Patients who were referred from the walk-in clinic setting directly to a hospital or emergency room for any procedure were excluded from analysis. Those patients in whom a trial of non-operative management failed before surgical treatment also were excluded.

Demographic data (age, gender, race, payor status, and injuries) were collected for all cohorts. The primary endpoints were number of days to initial evaluation by the treating pediatric orthopedic surgeon, number of days to definitive care, total number of providers seen during the episode of care, and total number of clinic visits required before surgery.

Comparative analysis was undertaken using descriptive statistics, and comparisons between cohorts were done using Student’s t-tests and Analysis of Variance (ANOVA). P-values less than 0.05 were considered significant.

## Results

A total of 162 patients with elbow or forearm injuries requiring surgery were identified from the medical record system. Ninety-eight patients who were evaluated initially in a walk-in clinic and subsequently had procedural or surgical treatment for an elbow or forearm injury were identified. Of these, 36 of 98 (37%) were initially evaluated by an orthopedic surgeon not fellowship-trained in pediatrics, and 62 of 98 (63%) were seen by a non-surgeon provider. The remaining 64 patients seen over the same time period at a regularly scheduled appointment and solely treated by a pediatric orthopedic surgeon served as the control group. Demographic information is listed in Table 1. There were no significant differences in age, gender, or race between these cohorts.

**Table 1:** Cohort Demographics

Demographics				
	Surgeon walk-in patients	Non-surgeon walk-in patients	Control group	P-value
Patients (n)	36	62	64	
Sex (n)				0.252
Male	21	41	33	
Female	15	21	31	
Mean age (years)	8.7 (1-17)	8.6 (4-16)	7.4 (1-14)	0.845
Race (n)				0.912
Caucasian	23	43	42	
African-American	10	15	19	
Other	3	4	3	

Non-surgeon versus orthopedic surgeon providers

No statistically significant differences were found between the two groups (non-surgeon versus orthopedic surgeon) of walk-in patients when comparing the time between initial evaluation and first visit with the treating pediatric orthopedic surgeon. Care of patients was transferred to a treating pediatric orthopedic surgeon an average of 3.9 days after initial walk-in evaluation by an orthopedic surgeon and 3.7 days by a non-surgeon provider (p = 0.631). Also, no significant differences were noted when comparing the time between initial walk-in evaluation and surgical treatment between the non-surgeon and surgeon groups. Walk-in patients seen by an orthopedic surgeon underwent surgery an average of 5.2 days after initial evaluation compared to 4.8 days for patients seen by a non-surgeon provider (p = 0.622). In both groups most of the patients had two visits with two different providers prior to their definitive procedure, with no significant differences noted between providers.

Control group

Sixty-four patients who were seen over the same time period at a regularly scheduled appointment and treated solely in a pediatric orthopedic surgeon’s clinic underwent a definitive procedure significantly faster than either walk-in group (3.3 vs. 5.2, p = 0.0004 and 4.8, p = 0.01, respectively). Additionally, patients solely seen by a pediatric orthopedic surgeon were significantly more likely to undergo their surgery after a single clinic visit. Twenty patients (56%) in the surgeon walk-in group and 24 patients (61%) in the non-surgeon walk-in group had two or more clinic visits prior to surgical treatment compared to the control group in which all 64 patients (100%) underwent their procedure after a single visit.

Analysis by injury type

In the walk-in group, distal radial and forearm shaft fractures were the most common diagnoses in 33 patients (34%) and 28 patients (29%), respectively. In contrast, the most common injuries seen in the pediatric orthopedist's group (control) were supracondylar fractures of the humerus (52%, 33 patients). The complete distribution of injuries is listed in Table 2. All supracondylar humeral fractures in this study were Gartland type II extension injuries.

**Table 2:** Injury Distribution

Injury Type	Walk-In	Control	
Distal Radius	33	Distal Radius	5
Forearm shaft	28	Forearm shaft	12
Supracondylar Humerus	19	Supracondylar Humerus	33
Medial epicondyle	6	Medial epicondyle	8
Lateral Condyle	5	Lateral Condyle	2
Radial neck	3	Radial neck	2
Monteggia	2	Monteggia	1
Olecranon	1	Coronoid	1
Galeazzi	1		

Patients with supracondylar fractures seen in the walk-in clinic underwent their procedures an average of 4.6 days after evaluation, compared to an average of 2.6 days for patients seen and treated by a pediatric orthopedic surgeon (p = 0.004). Times to treatment for the remaining diagnoses were not significantly different (Table 3).

**Table 3:** Analysis of Delay by Injury Type Sample sizes were too low for comparison for remaining diagnoses.

	Provider	N	Mean	Std. Deviation	P-value
Distal Radius	Walk-In	35	5.7	4.16	
	Same Surgeon	5	3.6	1.14	0.274
Forearm Shaft	Walk-In	28	4.46	2.99	
	Same Surgeon	12	4	2.3	0.634
Supracondylar	Walk-In	19	4.58	2.43	
	Same Surgeon	33	2.64	1.27	0.004
Medial Epicondyle	Walk-In	6	4.33	1.86	
	Same Surgeon	8	4.38	2.07	0.97

## Discussion

Demand for pediatric musculoskeletal care is high due in part to the fact that approximately 25% of the population in the United States is under 18 years of age and that this segment of the population has been shown to have increasing rates of musculoskeletal injury [10,11]. In addition to the high rates of traumatic injury, pediatric orthopedic surgeons also care for a wide variety of developmental, traumatic, and overuse syndromes that are commonly managed non-operatively. To meet this increasing demand, orthopedic practices across a variety of subspecialties have integrated non-surgeon physicians and advanced practice providers into the clinical care setting [1-5,9]. Many studies have demonstrated increasing enrollment in physician assistant and nurse practitioner programs, and the care they provide has been reported to be cost efficient and safe in a variety of clinical scenarios [5-9,12-14].

Advanced practice providers are increasing in orthopedic settings; however, data comparing the care they provide with that of orthopedic surgeons are lacking. A recent study by Althausen et al. analyzed the financial and clinical effects of adding a physician assistant to an orthopedic trauma service at a level-2 community trauma hospital [6]. They found that although physician assistants only covered approximately 50% of their costs, patients were seen faster and spent less time in the emergency department after physician assistant integration. Administration of antibiotics and venous thromboembolism prophylaxis were more reliable with physician assistants as part of the team, and overall postoperative complication rates were reduced. Additionally, multiple studies have found that advanced practice providers can improve the delivery of safe patient care in critical care, emergency, and general surgery settings [4-9,14-16].

This study directly compared the operative treatment of pediatric patients with forearm and elbow trauma initially seen by orthopedic surgeons and non-surgeon care providers in a large, single-specialty orthopedic walk-in clinic. No significant differences between surgeon and non-surgeon providers were found in days before appointments with the treating pediatric orthopedic surgeon or total days elapsed between clinic evaluation and definitive surgery. This suggests that advanced practice providers can be safely integrated into orthopedic clinical treatment teams without introducing a significant delay in care.

Although no differences were found when comparing orthopedic surgeons with non-surgeon providers in the walk-in setting, patients seen and treated initially by a pediatric orthopedic surgeon underwent their procedures significantly sooner than patients seen in the walk-in clinic. This may be attributable to the pediatric surgeon’s ability to diagnose the injury and schedule surgery in a single encounter. Additionally, the control group contained a higher proportion of supracondylar fractures, and these injuries were treated significantly faster by the pediatric orthopedic surgeon.

Aside from being retrospective in nature, this study has other limitations, and our findings may not be generalizable to advanced practice providers seeing patients with musculoskeletal complaints in non-orthopedic clinics. This study was completed at a high-volume, academic orthopedic practice where non-surgical providers are well integrated into the treatment team. Non-surgical team members can easily communicate with orthopedic surgeons either in person or by telephone, which is unlikely to be the case in smaller orthopedic practices, multi-specialty clinics, or urgent care facilities. The results of this study are most applicable to larger orthopedic groups with multi-specialty representation. Also, selection bias potential exists as a result of excluding patients who were prescribed a trial of non-operative management because they may represent the group with the most variability in treatment between different types of providers. Larger, prospective studies will be needed to better assess patient care by advanced practice and non-surgeon providers as their participation in the orthopedic workforce continues to expand.

## Conclusions

In this retrospective study of 162 pediatric patients with upper extremity injuries, the time from initial evaluation to definitive treatment was assessed under three different outpatient scenarios: initial evaluation by a non-surgeon provider in a walk-in clinic with subsequent referral for definitive treatment; initial evaluation by an orthopedic surgeon in a walk-in clinic with subsequent referral for definitive treatment; and initial evaluation with definitive treatment by a fellowship-trained pediatric orthopedic surgeon at a regularly scheduled office appointment. No significant differences were found between the non-surgeon providers and orthopedic surgeons in the walk-in clinic, suggesting that advanced practice providers do not introduce significant delays in care. A statistically significant difference was noted between the walk-in clinic (regardless of provider) and the regularly scheduled office appointment; however, this delay of just over 1 day may not be clinically significant.
